# Subclinical Avian Influenza A(H5N1) Virus Infection in Human, Vietnam

**DOI:** 10.3201/eid1910.130730

**Published:** 2013-10

**Authors:** Mai Quynh Le, Peter Horby, Annette Fox, Hien Tran Nguyen, Hang Khanh Le Nguyen, Phuong Mai Vu Hoang, Khanh Cong Nguyen, Menno D. de Jong, Rienk E. Jeeninga, H. Rogier van Doorn, Jeremy Farrar, Heiman F.L. Wertheim

**Affiliations:** National Institute of Hygiene and Epidemiology, Hanoi, Vietnam (M.Q.Le, H.T. Nguyen, H.K.L. Nguyen, P.M.V. Hoang, K.C. Nguyen);; Wellcome Trust Major Overseas Programme, Hanoi and Ho Chi Minh City, Vietnam (P. Horby, A. Fox, M.D. de Jong, H.R. van Doorn, J. Farrar, H.F.L. Wertheim);; Centre for Tropical Medicine, Oxford, United Kingdom (P. Horby, A. Fox, H.R. van Doorn, J. Farrar, H.F.L. Wertheim);; Academic Medical Center, Amsterdam, the Netherlands (M.D. de Jong, R.E. Jeeninga)

**Keywords:** avian influenza A(H5N1), influenza, avian influenza, H5N1, Vietnam, human, subclinical, viruses, asymptomatic, clades, poultry

## Abstract

Laboratory-confirmed cases of subclinical infection with avian influenza A(H5N1) virus in humans are rare, and the true number of these cases is unknown. We describe the identification of a laboratory-confirmed subclinical case in a woman during an influenza A(H5N1) contact investigation in northern Vietnam.

In 2012, a debate was published in Science about the number of humans who have experienced subclinical infection with avian influenza A H5 and how this unknown denominator could affect the case-fatality rate reported by the World Health Organization (*1**,**2*). The controversy rests, to a large extent, on interpretation of serologic tests used to detect prior H5 infection and the paucity of virologically confirmed subclinical or mild cases. Here we describe a case of subclinical avian influenza A H5 infection, confirmed both virologically and serologically.

## The Case

The subclinical case was detected in 2011 during a contact investigation of a 40-year-old man suspected of having influenza A(H5N1) virus infection. The man’s household had sick poultry that were consumed by household members. The chickens roamed close to the sleeping area of the household members. The index case-patient, his daughter, and his daughter-in-law were involved in slaughtering and preparing the chickens. The index case-patient had fever, cough, dyspnea, and diarrhea that progressed over 2 days, leading to hospital admission. Despite intensive care and treatment with oseltamivir and antibiotics, the disease progressed, and he died 2 days later.

A throat swab taken from the index case-patient on day 3 of illness was tested by reverse transcription PCR, and results were positive for influenza A(H5N1) virus. Hemagglutination inhibition (HI) and microneutralization (MN) tests for H5N1-specific antibodies were negative in samples taken during the acute phase of illness (Technical Appendix).

On day 5 of illness of the index case-patient, a contact investigation was initiated. Throat swab specimens were collected from 4 household members and 1 close contact of the index case-patient: his spouse (age 47 years), daughter (age 18 years), daughter-in-law (age 25 years), and grandson (age 1 year) and an unrelated man (age 43 years). None of the contacts had signs or symptoms. Infection control measures were initiated, and all household members were given oseltamivir (75 mg/d) for 1 week and instructed to seek immediate health care if fever or respiratory symptoms developed. Results of HI testing of serum samples collected during the acute illness phase of the index case-patient were negative.

The human throat swab samples were tested by conventional RT-PCR. The sample from the index case-patient’s daughter, collected 6 days after the woman had slaughtered a chicken, was positive for influenza A/H5 by real-time RT-PCR, and virus was recovered on day 10 of inoculation on MDCK cells (Technical Appendix). The virus was identified by sequencing as influenza A/H5, clade 2.3.2.1. The woman had no signs or symptoms at the time the throat swab was collected, nor did she report any symptoms to health authorities during the subsequent week. Chickens were also tested, and 4 chickens in the commune tested positive for influenza A(H5N1) virus by RT-PCR of throat and cloacal swab specimens.

Repeat throat swab specimens collected from the 4 household contacts 6 days after the initial collection yielded negative test results for influenza/H5. Serologic testing showed seroconversion only in the woman with subclinical infection; her HI titer increased from <20 to 160 against both clade 2.3.4 and 2.3.2.1 viruses (Table 1). During a second contact investigation 1 month later, 20 other members of the commune were screened by RT-PCR of throat swab specimens and serologic testing. All results were negative for influenza A(H5N1) virus.

**Table 1:** HHI and MN assay titers for serum samples from woman with subclinical influenza A(H5N1) virus infection, Vietnam, 2011

Clade and sample	HHI	MN
Clade 1		
First sample, day 5	<20	ND
Second sample, day 11	40	<10
Third sample, day 41	40	<10
Clade 2.3.2.1		
First sample, day 5	<20	ND
Second sample, day 11	80	80
Third sample, day 41	160	160
Clade 2.3.4		
First sample, day 5	<20	ND
Second sample, day 11	80	40
Third sample, day 41	160	40

*Sample days are days since disease onset in index case-patient. HHI, horse hemagglutination inhibition; MN microneutralization; ND, not done.

The full genome of the identified virus strain (A/CM32/2011) was sequenced (Technical Appendix) and confirmed to be clade 2.3.2.1 by using the Highly Pathogenic H5N1 Clade Classification Tool (*3*). The open reading frames of the genes were translated and aligned with all clade 2.3.2.1 sequences available from the Influenza Research Database (www.fludb.org). Amino acid changes are summarized in Table 2. A phylogenetic analysis of clade 2.3.2.1 from Vietnam sequences showed a high homology between the samples, including A/CM32/2011 (Figure). To identify possible changes specific to human infection, the differences between the clade consensus and A/CM32/2011 were compared with influenza A(H5N1) virus samples from Asia. No amino acid changes were preferentially seen in human samples compared with the avian samples. A/CM32/2011 contains the N170D (*4*) and T172A mutations in HA that are associated with airborne transmissibility of influenza A(H5N1) virus in ferrets (*5*); these mutations are found in most avian influenza A(H5N1) clade 2.3.2.1 samples (Table 2, Appendix). The results indicate that the virus is a typical influenza A(H5N1) clade 2.3.2.1 virus, with no remarkable changes.

**Table 2:** Amino acid changes detected in first passage of full-genome sequence analysis of influenza A(H5N1) virus isolate from subclinical human infection, Vietnam

Gene and mutation	Mutation frequency, %
Polybasic 2, n = 112	
S107N	15
I261T	2
K312R	15
Nucleoprotein, n = 125	
I33V	48
H52Y	50
R77K	45
T395N	49
Polymerase, n = 51	
A20T	12
D27S	12
V63A	29
T85T	33
K142R	12
Y241C	45
Q259P	33
L261M	29
A263T	43
D272E	12
V323I	2
E352D	12
R353K	29
R391K	29
S400P	35
A404S	29
D547N	2
A552T	37
N614T	29
G631S	29
S648C	2
L711I	2
Polybasic 1, n = 129	
K54E	22
S158N	49
T182I	2
N314S	2
D383E	30
L384I	7
I525V	9
S633N	2
K635R	19
Hemagglutinin, n = 244	
T9A	49
L82M	36
S139T	6
S152P	36
D199N	2
F455Y	1
K461R	8
Neuraminidase, n = 177	
I8V	16
V13I	27
I16V	21
A46T	14
T64A	1
A66S	1
H106N	1
M107L	23
V143I	10
T168I	2
G200R	49
A212T	1
S265A	36
T312E	1
M318V	26
I326V	21
N346H	13
V369M	19
Nonstructural 1, n = 145	
S73P	11
D87E	6
M118I	28
V123I	28
E148K	1
S166G	25
M217L	1
T220A	6

**Figure:**
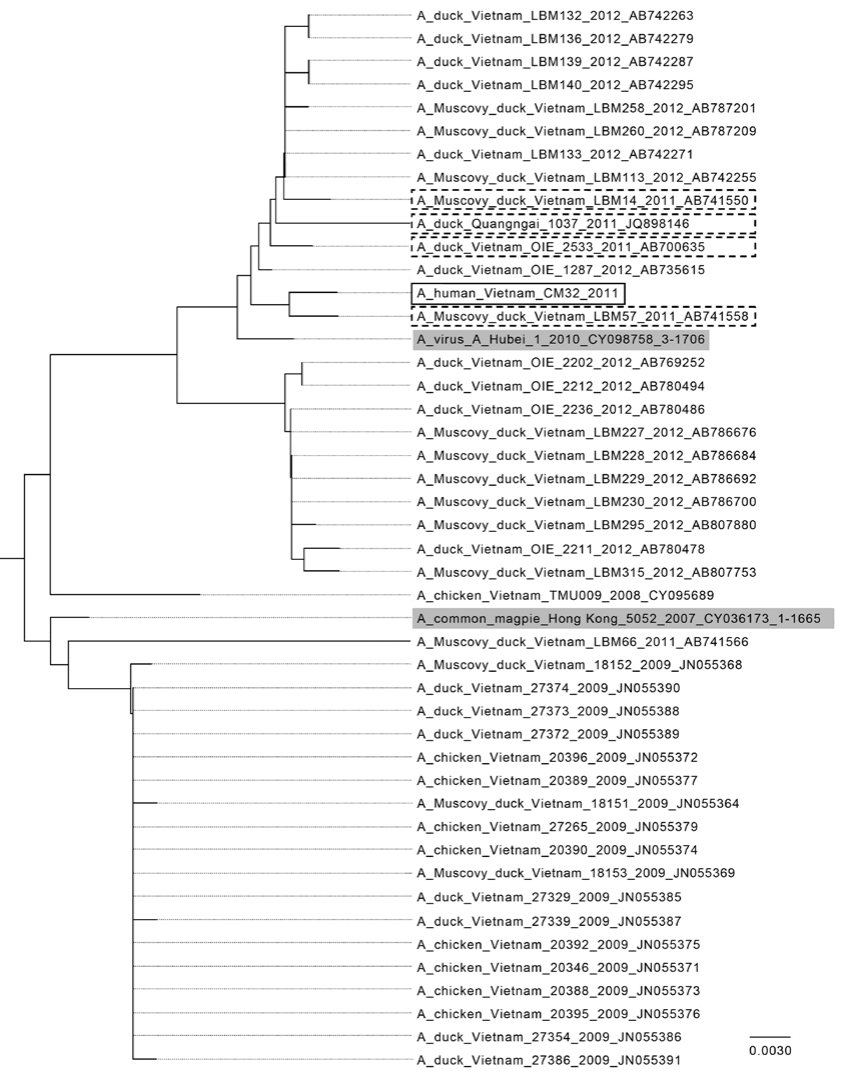
Phylogentic analysis of avian influenza A(H5N1) virus clade 2.3.2.1 hemagglutinin DNA sequences from Vietnam compared with other isolates. Solid black box indicates isolate from the subclinical human case investigated in this study, A/CM32/2011; dashed boxes indicate sequences from Vietnam in 2011; gray shading indicates World Health Organization vaccine candidates A/common magpie/Hong Kong/5052/2007 and A/Hubei/1/2010 for clade 2.3.2.1. The sequences were downloaded from the Influenza Research Database (www.fludb.org), imported into MEGA 5.2 (www.megasoftware.net), and aligned by using MUSCLE (EMBL-EBI, Cambridgeshire, UK). The neighbor-joining tree was generated from the aligned sequences using standard settings. Scale bar indicates nucleotide substitutions per site.

## Conclusions

We report subclinical infection with avian influenza A(H5N1) virus in a human in Vietnam, confirmed by RT-PCR, virus isolation from throat swab, and detection of specific antibodies. A subclinical case was also reported from Pakistan in 2008 (*6*). Sequence analysis of the Vietnam case showed that the infecting virus belonged to influenza A(H5N1) clade 2.3.2.1. This clade was first detected in poultry in northern Vietnam in early 2010 and replaced clade 2.3.4 in that area, whereas clade 1 remains predominant in southern Vietnam, with 4 confirmed cases reported in early 2012 (*7*). The recent clade 2.3.2.1 has evolved from clade 2.3.2 viruses that has circulated among poultry in eastern Asia since 2005 and has become predominant in several Asian countries. Since clade 2.3.2.1 viruses were initially detected in Vietnam, prevalence has increased in poultry, but no associated rise in detection of human cases has been observed (*7*). Similarly, clade 2.3.2.1 virus has been circulating in poultry in India, but no human cases have been reported (*8*). The HA sequence of this virus is similar to an influenza A(H5N1) virus detected by RT-PCR in a 3-year-old patient with influenza-like illness investigated as part of the National Influenza System Surveillance in 2010 (M.Q. Le, unpub. data). This patient had mild symptoms and survived, which raises the possibility that this strain represents a less virulent form of influenza A(H5N1) in humans.

In our investigation, the case-patient with subclinical infection was treated with oseltamivir while she was asymptomatic, which may explain why she did not develop clinical disease. Studies using human volunteers indicate that seasonal influenza virus shedding may occur ≈24 hours before symptom onset in 25%–30% of patients (*9*). Likewise, community cohort studies show presymptomatic shedding and asymptomatic shedding in 15%–20% of patients infected with seasonal influenza viruses and with influenza A(H1N1)pdm09 virus (*10**–**12*). Oseltamivir is known to prevent disease when given before inoculation in human volunteers and to shorten duration and lessen the severity of illness in natural infection (*13*), but we found no evidence in clinical and volunteer studies from the literature suggesting that oseltamivir may prevent clinical illness once detectable infection has been established, as we found in this subclinical case (*13**,**14*). The patient we investigated probably was exposed during slaughtering of a chicken 6 days before her positive throat swab was collected. However, because chickens in the commune tested positive at the time of the contact investigation, ongoing exposure to influenza A(H5N1) cannot be excluded as the source of infection. Furthermore, the patient may not have reported symptoms to the health authorities for personal reasons.

Thus far, evidence of subclinical influenza A(H5N1) virus infections has been collected on the basis of serologic testing only (*1*), but it is unclear whether serologic testing reliably detects subclinical cases. According to the World Health Organization, MN titers >80 are indicative of infection but must be confirmed by a second serologic test because of the possibility of cross-reactivity (*1*). The interpretation of results from a single serum sample is limited by the specificity or sensitivity of serologic tests, and viral shedding times may mean that infected cases may be missed. Estimating the incidence of asymptomatic influenza A(H5N1) virus infection in humans exposed to sick poultry or human case-patients requires further careful study using early collection of swab samples and paired acute and convalescent serum samples.

Technical Appendix Detailed methods of isolation and sequencing for influenza A(H5N1) virus from subclinical human case, Vietnam.
