# Supplementary material for: Subclinical Avian Influenza A(H5N1) Virus Infection in Human, Vietnam
Source: Emerg Infect Dis. 2013 Oct;19(10):1674–7. doi: 10.3201/eid1910.130730 (PMC3810763; doi:10.3201/eid1910.130730)
Supplement: Technical Appendix — Detailed methods of isolation and sequencing for influenza A(H5N1) virus from subclinical human case, Vietnam. [file 13-0730-Techapp-s1.pdf]

Article DOI: http://dx.doi.org/10.3201/eid1910.130730

Subclinical Avian Influenza A(H5N1) Virus Infection in Human, Vietnam

Technical Appendix

Detailed Methods

Influenza Isolation

A 100 µL sample inoculated onto MDCK cells (Madin-Darby canine kidney cells-American Type Culture Collection-ATCC, USA), which were maintained in minimum essential medium (MEM) supplemented with 5% newborn calf serum (Sigma, St. Louis, MO, USA) and antibiotics at 37°C in 5% CO_2. All experiments were performed in a biosafety level 3 containment laboratory at NIHE, Hanoi. MDCK cells were checked daily for cytophathic effects (CPE) and virus was harvested and stored in −80 C when CPE reached 80%.

Serology

Haemagglutination inhibition (HI) assays were performed as described (*1*) by using receptor-destroying enzyme (RDE) treated patient and reference sera at a starting dilution of 1:20, 8 HA units of virus (in 50 µL), and 1% v/v horse erythrocytes. The viruses, inactivated by 1% β propiolactone (BPL), used were: A/Vietnam/HN 30408/2004, A/HN 30850/2005, and A/CM32/2011, representing clade 1, clade 2.3.4, and clade 2.3.2.1, respectively. HI titers were read up to 60 minutes after the addition of erythrocytes and reported as the reciprocal of the highest serum dilution causing complete inhibition of agglutination.

Microneutralization (MN) assays were performed according to WHO protocols, using 100 x $TCID_{50}$ of live viruses as above (*2*). Virus was incubated with 2-fold serial dilutions of sera starting at 1:10 and then incubated with MDCK cells overnight before virus quantitation by ELISA to detect influenza nucleoprotein. The titer was reported as the reciprocal of the highest dilution that reduced infection by at least 50%.

Sequencing

The RNA was isolated from the virus (1st passage) isolated from the subclinical case and used for an8-segment RT-PCR to generate the full length 8 influenza segments as described previously (*3*). The RT-PCR reaction was performed with primers common-uni12R (5′-GCCGGAGCTCTGCAGATATCAGCRAAAGCAGG-3′), and common-uni13 (5′-CAGGAAACAGCTATGACAGTAGAAACAAGG-3′) using the One-Step RT-PCR kit High Fidelity (Invitrogen, NY, USA). The cDNA was sheared (Adaptive Focused Acoustics, Covaris, USA) to yield fragments ranging from 300 to 1000 bp and y-primers from the Rapid library 454 kit (Roche, USA) were ligated to the fragments with use of an automated SPRI works Fragment Library System II (For Roche GS FLX* DNA Sequencer) according to the manufacturer's protocol (Beckman Coulter, CA, USA). The quantity of properly ligated fragments was determined based on the incorporation efficiency of the fluorescent primers using a FLUOstar OPTIMA (BMG Labtech, Germany). Emulsion PCR, bead recovery and enrichment were performed manually according to the manufacturer's protocol. The sample was sequenced by the Laboratory of Genome Analysis at the Academic Medical Center (Amsterdam, The Netherlands).

The resulting reads were analyzed as described before (*3*) and used to generate a consensus sequence for each segment. Since the coverage of the larger segment was relatively low, an additional PCR was performed for the PA, PB1 and PB2 segments and sequenced with standard Sanger sequencing using degenerate primers for H5N1 (*4*). The resulting consensus sequences were analyzed with the blast and H5N1 clade identifier at IRD site (*5*). All available clade 2.3.2.1 sequences were compared with the sample sequence. The differences between the samples and the clade 2.3.2.1 consensus were subsequently compared to Asian avian human H5N1 samples.

References

1. Stephenson I, Wood JM, Nicholson KG, Zambon MC. Sialic acid receptor specificity on erythrocytes affects detection of antibody to avian influenza haemagglutinin. J Med Virol. 2003;70:391–8. PubMed http://dx.doi.org/10.1002/jmv.10408

2. World Health Organization. Manual for the laboratory diagnosis and virological surveillance of influenza. Geneva: World Health Organization; 2011.

3. Watson SJ, Welkers MR, Depledge DP, Coulter E, Breuer JM, de Jong MD, et al. Viral population analysis and minority-variant detection using short read next-generation sequencing. Philos Trans R Soc Lond B Biol Sci. 2013;368:20120205. PubMed http://dx.doi.org/10.1098/rstb.2012.0205

4. Herfst S, Schrauwen EJ, Linster M, Chutinimitkul S, de Wit E, Munster VJ, et al. Airborne transmission of influenza A/H5N1 virus between ferrets. Science. 2012;336:1534–41. PubMed http://dx.doi.org/10.1126/science.1213362

5. Squires RB, Noronha J, Hunt V, Garcia-Sastre A, Macken C, Baumgarth N, et al. Influenza research database: an integrated bioinformatics resource for influenza research and surveillance. Influenza Other Respi Viruses. 2012;6:404–16. PubMed http://dx.doi.org/10.1111/j.1750-2659.2011.00331.x
